# 529. Systematic Review and Meta-Analysis of Ivermectin Safety Profile in COVID-19 Trials

**DOI:** 10.1093/ofid/ofab466.728

**Published:** 2021-12-04

**Authors:** Hannah Wentzel, Junzheng Wang, Abbienaya Dayanamby, Victoria Pilkington, Jacob Levi, Andrew Hill, Leah Ellis

**Affiliations:** 1 Imperial College, London, UK; 2 University of Oxford, Oxford, UK; 3 University College London Hospitals, London, UK; 4 University of Liverpool, London, UK; 5 Imperial College London, London, UK

## Abstract

**Background:**

There is a continued and pressing need for safe and effective treatment of COVID-19. Significant survival benefits have been shown by dexamethasone, tocilizumab and sarilumab, however they are only recommended in hospitalised COVID-19 patients. Ivermectin is a well-established and readily available antiparasitic drug which may be suitable for treatment in mild and moderate disease stages. It recently demonstrated anti-viral properties *in vitro* and now over 80 clinical trials have been registered worldwide to test its effectiveness in COVID-19 patients. This meta-analysis aims to collect data on adverse events reported in new COVID-19 treatment trials for the use of ivermectin as a repurposed medication.

**Methods:**

Data was extracted from randomised trials of COVID-19 treatment trials identified through systematic searches of PUBMED, EMBASE, MedRxiv and trial registries. The primary outcome of this meta-analysis is the frequency of adverse events. Key safety events included serious, gastrointestinal, neurological, cardiovascular and dermatological adverse events.

**Results:**

Overall, 18 trials investigating ivermectin for COVID-19 in a total of 2496 participants reported safety data and were included. There was no significant difference in the proportion of all adverse events between ivermectin and the comparator. There were 371/1261 (29%) adverse events recorded in the ivermectin containing arms and 376/1284 (29%) in the control arms (RR 1.02 [95% CI 0.77 - 1.34]; p = 0.91). There was no significant difference in the rate of serious adverse events across treatment arms (RR 1.95 [95% CI 0.75 - 5.11]; p = 0.18). No significant differences between ivermectin and the control were seen across different subcategories of adverse events. Figure 1 shows a summary of the results for all adverse events.

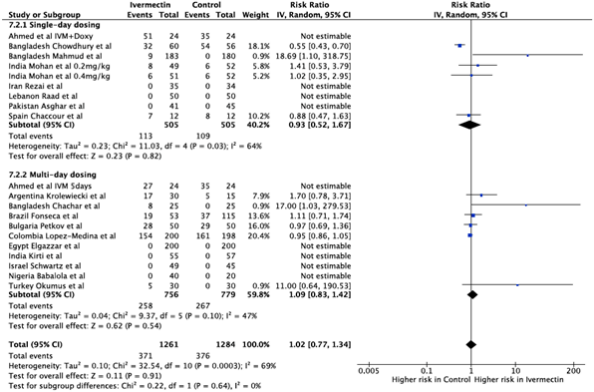

Forest plot comparing ivermectin and the control for all adverse events in COVID-19 trials, subdivided into single-day dosing trials and multi-day dosing trials.

**Conclusion:**

The results of recent COVID-19 trials show that overall, ivermectin is safe and well-tolerated. No significant difference in adverse event reporting was found across all subgroups in single and multi-day treatment regimens for the studies analysed. Safety reporting methodologies often varied across trials. Future and ongoing trials should be encouraged to collect and monitor safety data systematically.

**Disclosures:**

**All Authors**: No reported disclosures

